# Adolescents’ Perspectives on a Mobile App for Relationships: Cross-Sectional Survey

**DOI:** 10.2196/mhealth.8831

**Published:** 2018-03-08

**Authors:** Bridianne O'Dea, Melinda Rose Achilles, Aliza Werner-Seidler, Philip J Batterham, Alison L Calear, Yael Perry, Fiona Shand, Helen Christensen

**Affiliations:** ^1^ Black Dog Institute University of New South Wales Randwick Australia; ^2^ Centre for Mental Health Research Australian National University Canberra Australia

**Keywords:** family relations, peer group, help-seeking behavior, mobile apps, adolescence

## Abstract

**Background:**

Adolescence can be a fertile time for relationship issues, with interpersonal conflict being a risk factor for poor mental health. Mobile app interventions may have a significant appeal to young people in assisting with relationship distress. However, currently available apps have not been formally evaluated. Youths’ perspectives on engaging with mobile technology to assist with relationships are also unknown.

**Objective:**

This study aimed to examine adolescents’ attitudes toward the concept of a mobile phone app for relationship help and support, and whether they would be likely to use such an intervention.

**Methods:**

A cross-sectional Web survey consisting of 42 questions, including 13 free responses, was delivered. The proposed app, including character vignettes, was presented, and participants were asked to indicate whether they had experienced the same relationship issues, whether their peers would relate to the issues, and how helpful they found the proposed advice. Participants were also asked to provide their own suggestions for help, which were analyzed using thematic analyses.

**Results:**

A total of 150 adolescents (aged 15 to 18) participated. Overall, 60.7% (91/150) were likely to use an app for relationship problems, and this was not associated with demographics or social support (all *P* values >.05). Likelihood of app usage was found to be influenced by perceived need for help, personal beliefs about app effectiveness, and whether the app is engaging and easy to use. Overall, adolescents were receptive of the proposed content with an average of 99.3% (149/150), rating the strategies provided as somewhat to very helpful.

**Conclusions:**

Adolescents were likely to use a mobile phone app for relationship support, and use was not influenced by gender, age, social support, or any other background characteristic. Instead, likely use was influenced by need, personal beliefs, usability, and the appropriateness of app content. App developers must address these factors if the app is to have a wide-scale uptake.

## Introduction

Across the lifespan, relationships are important to well-being [1]. Relationships generate social support that helps individuals to buffer psychological distress and prevents maladaptive coping [2,3]. Positive relationships are highly protective against a range of poor health outcomes [4], including mental illness [5]. Adolescence is an active phase of relationship development [6-8]. During this time, young people manage the desire for peer interaction and approval, with an increasing independence from the family. Young people begin to establish relationships on shared values, ideas, and intimacy rather than the convenience and common interests, which characterize childhood friendships [9]. Adolescence can be a fertile time for relationship problems, and although expected, some can be disruptive and distressing. One in 5 youths is concerned about the level of interpersonal conflict in their life [10]. Up to 25% have reported experiencing recent psychological distress because of a family or interpersonal issue, and rates were higher among females [11]. Relationship problems can elevate the risk of suicide [12,13], depression [14], anxiety [15], school disengagement [16], substance misuse [17], and poor physical health [18]. Given these negative consequences, it is important to ensure young people are adequately supported when faced with relationship distress.

### Help-Seeking for Relationships

In general, little is known about adolescents’ help-seeking specifically for relationships. Much more is known about adolescents’ help-seeking for mental health issues, which has been found to be inhibited by stigma, accessibility, and self-reliance [19]. Conversely, positive past experiences, social support, and encouragement from others is found to aid help-seeking. Although mental health issues are more complex, similar factors may affect help-seeking for relationships. Many adolescents report feeling embarrassed and ashamed of personal issues [20], believing relationship problems to be significantly more intimate than other types of problems, such as physical health, education, finance, or legal issues [21]. Help-seeking for relationships may be complicated by young people’s preferences for turning to friends and family [22,23]. If these sources have been compromised because of conflict, the capacity of the support network may be diminished, and help-seeking may be inhibited. In a study by Boldero and Fallon [11], interpersonal problems were found to be associated with greater help-seeking when compared with family problems, despite family problems being more frequently reported [11]. Young people perceived interpersonal problems to be more serious than those with family, and that their locus of control over interpersonal problems was greater. Help-seeking was predicted by gender, and problem type, with females more likely to report problems with families and interpersonal relationships, whereas males were more likely to report problems with education [11]. Combined, past studies depict a complex picture of the factors influencing help-seeking among youths.

### Mobile Apps for Relationships

There are evidence-based therapies (eg, cognitive behavioral therapy [CBT] and interpersonal therapy [IPT]) that have been found to be effective for relationships [24-26]. These types of interventions are delivered by trained professionals and are typically conducted over a series of weekly sessions. Despite the effectiveness, uptake among youths is likely to be low because of limited financial capacity and a reluctance of formal help [20,22,23]. CBT has been adapted for Internet delivery in the form of self-directed programs, overcoming many of the access barriers. However, these programs are designed to treat symptoms of depression and anxiety rather than prevent relationship distress. Consequently, these programs may not appeal to youths who are seeking help specifically for relationships. A recent systematic review found that mobile health interventions are a viable health behavior change modality for youths [27]. A benefit of mobile help-seeking interventions is that they can deliver brief and engaging content that has the potential to prevent mental health issues, without pathologizing normal relationship patterns. Although there are some mobile apps currently available for relationships, they predominately focus on relationship separation and neglect the range of other relationship issues young people face, for example, family conflict and psychosocial experiences [28,29]. These apps also primarily use one type of therapeutic intervention, behavioral activation, in which the user is encouraged to schedule pleasurable activities with others [30]. Few of these apps have been formally evaluated. Therefore, there is limited evidence to support the use of currently available interventions.

### Our Study

Given the emotional impact of relationship conflict, the lack of help being sought for these issues, and the preference for digital health solutions, there is a clear need for an evidence-based mobile app that has universal appeal, covers a range of relationship types (eg, friendships, family relationships, romantic relationships) and psychosocial issues (eg, anxiety, body image, negative thinking, help-seeking), and is easily accessible to youths. Researchers at the Black Dog Institute have developed the content for such an app. However, to ensure that the proposed app has uptake, acceptability needs to be assessed. In the context of health care, acceptability is defined as a multifaceted construct that reflects the extent to which people delivering or receiving an intervention consider it to be appropriate, and suited to their needs, based on anticipated or experimental responses [13]. As outlined by their review, Sekhon et al [31] argue that acceptability should be assessed before individuals engage in an intervention and should measure how a person feels about an intervention, the extent to which the participant understands the intervention content, the extent to which the intervention is perceived as likely to achieve its purpose, and the participants’ confidence in using the intervention. Guided by this framework, this study aimed to examine young people’s attitudes toward the concept of a mobile phone app for relationship help and support, and whether they would be likely to use such an intervention. Using a series of vignettes designed to be incorporated into the app’s content, this study aimed to examine the following: (1) whether young people had experienced the types of relationship issues presented; (2) the extent to which young people felt their peers would relate to the proposed content; and (3) the level of acceptability of the relationship strategies offered by the app. The study also examined whether acceptability was influenced by demographic factors or social support levels. Results will help to highlight which aspects of the proposed content could be modified to increase acceptability, and thus participation. This study presents a systematic approach to understanding end users’ needs [32,33], which will support the future development of engaging and effective mobile help-seeking interventions.

## Methods

### Design

A cross-sectional Web survey was delivered. This study was approved by the University of New South Wales Human Research Ethics Committee (#HC15583).

### Participants and Procedure

Australian adolescents aged 15 to 18 years were invited to participate in the study by responding to an online advertisement, which included a link to the online survey published on Facebook as well as the Black Dog Institute’s website, Facebook, Twitter, and Instagram platforms. The online survey was delivered by the Key Survey software, hosted by the University of New South Wales. The survey included the participant information sheet and consent form in which participants were asked to provide consent online. Parental consent was not required, as young people aged 15 years and over were deemed to be mature minors capable of consenting to their own participation in this low-risk research. Once consent was given, the survey questions appeared. After completion of the survey, participants were redirected to a separate webpage on which they were asked to enter their name and email address to be reimbursed with an Aus $20 online gift voucher. Personal details were not linked to survey responses.

### Survey

The survey consisted of 42 questions, including 13 free-response questions.

#### Demographics

A total of 7 questions were asked, assessing age, gender (male, female, or other), country of birth (Australia or other), language spoken at home (English or other), living situation (with both parents together or all other), work and study status (high school, working, university, apprenticeship, or none of the above), and whether they identified as lesbian, gay, bisexual, trans, intersex (LGBTI; answered yes, no, or rather not say) or Aboriginal and, or, Torres Strait Islander (ATSI; answered yes, no, or rather not say). Participants were also asked whether they owned a smartphone (yes or no) or mobile tablet (yes or no) and the primary device used to access the Internet.

#### Social Support

To determine whether the acceptability of the app was associated with current social support levels, the Schuster Social Support Scale [34] was used to measure the extent of positive and negative social interactions in 3 domains: peers, family, and partner. The scale consists of a total of 15 items: 5 items related to participants’ peers (2 positive and 3 negative), 5 items (2 positive and 3 negative) to participants’ family, and 10 items (5 positive and 5 negative) to participants’ partner, where applicable. Participants responded using a Likert scale with 4 possible degrees of agreement, ranging from 1 to 4 (*How often…* questions answered never, rarely, sometimes, often and *How much…* questions answered not at all, a little, some, or a lot). Positive and negative subscale scores for each source of support were calculated by adding each item score and then dividing by the total number of items. Higher scores reflect higher levels of positive or negative support.

#### Likelihood of App Usage

Participants were asked how likely they were to use a mobile phone app for relationships (answered likely, neutral, or unlikely). Using free response, they were also asked to provide reasons why or why not, which were to be analyzed qualitatively.

#### Acceptability of App Content

The survey included 4 nonstandardized character vignettes that each described relationship issues experienced by 4 young people named Abigail, Jasper, Emily, and Angus. Outlined in Table 1, these vignettes were created specifically for this study by mental health researchers and clinicians and were designed as potential characters to be included in the app for the purposes of social learning. Each vignette was approximately 250 words in length and had a Flesch-Kincaid Grade Level score of 6, indicating a Grade 6 reading level. Vignettes are provided in Multimedia Appendix 1.

Participants were asked to read the vignettes and report whether they or a friend had experienced this situation (experience answered yes, no, or not sure). They were then asked to rate how much they felt their peers would relate to the character (relatedness answered not at all, a little, moderately or a lot). Given that the aim was to design a universal app with broad appeal, the relatedness variable was collapsed (a lot, moderately, a little vs not at all) to better capture what types of youths did not at all relate to the content. Using free response, participants were asked to report what they would do if faced with the character’s issue, and what they would suggest a friend do in a similar situation. Finally, evidence-based coping strategies were presented. Participants were then asked to rate the helpfulness of these using a 5-point scale of not at all helpful (1) to extremely helpful (5).

**Table 1:** Relationship issues and coping strategies outlined in the character vignettes.

Character	Issues explored	Coping strategies
Abigail	Peer conflict, intimate relationship problems, eating disorders, negative thinking, and low self-esteem	Coping with distress, help-seeking, and relaxation and meditation
Jasper	Relationship breakdown, social anxiety, bullying, online relationships, and low self-esteem	Problem solving, sleep strategies, anxiety desensitization, and help-seeking
Emily	Academic pressure, parental conflict, peer conflict, drug use, sexuality, and negative thinking	Help-seeking, relaxation, cognitive restructuring, and social mapping
Angus	Family conflict, parent separation, intimate relationship problems, anger management, substance use, and change	Help-seeking, relaxation, conflict resolution, and cognitive restructuring

There were no minimum levels of experience, relatedness, or helpfulness expected. Instead, the study aimed to identify the aspects of the app content that may influence acceptability.

### Analysis

The data were exported from Key Survey, and statistical analyses were conducted in SPSS v22 (Chicago, IL, USA). Descriptives were conducted and reported. Correlational and chi-square tests were used to examine whether background factors were associated with having experienced a character’s issues, peer relatedness, and helpfulness ratings. This would help to determine whether the app content was more likely to be acceptable among certain youths. Due to the low cell counts, participants who reported that their gender was other (n=5) or that they would rather not report their LGBTI status (n=3) were excluded. Furthermore, this sample was inappropriate for examining ATSI effects because of low numbers (2.0%; 3/150), and the saturation of technology ownership (98.0%; 147/150) meant that these variables were inappropriate for inclusion in the variance analyses. For the analyses including the experience variable, participants who reported that they were unsure were excluded. This was done to ensure integrity of the data. Free-response data were analyzed using Braun and Clarke’s thematic analysis guidelines [35]. The analysis involved manually coding the 13 free-response questions. Using an inductive approach, patterns and themes were identified. Two researchers (BOD and MA) refined the initial codes for cohesiveness, sorted to combine related concepts into encompassing main themes, and reached an agreement on the final themes. Using the predetermined framework, the data were then reviewed by a third researcher (YP). The mean intercoder reliability between the 2 coders (YP and MA) was 77% (range: 70%-83%). Inconsistencies were identified and resolved using consensus. In accordance with recommendations [36,37], frequency counts and percentages were reported to highlight the representativeness of themes and clarify meaning inferred from the dataset.

## Results

### Participants

A total of 150 adolescents completed the survey (age range: 15-18 years, mean 16.8 years [SD 1.1]). Table 2 outlines participant characteristics.

Table 3 presents the reported levels of social support. Overall, participants had higher positive support than negative support in each domain. Results show that family members and partners provided slightly higher positive support compared with participants’ peers. Family members also provided the highest level of negative support across the domains.

**Table 2:** Participant characteristics (N=150).

Demographic		n (%)
Female		104 (69.3)
Born in Australia		130 (86.7)
English is main language		138 (92.0)
LGBTI^a^		38 (25.3)
ATSI^b^		3 (2.0)
At high school		98 (65.3)
At university		39 (26.0)
Working full-time		6 (4.0)
Living with both parents together		95 (63.3)
In a relationship		44 (29.3)
Owned a smartphone		147 (98.0)
Owned a tablet		92 (61.3)
Owned both		90 (60.0)
Did not own either		1 (0.7)
**Device mainly used to access Internet**		
	Personal laptop or desktop computer	77 (51.3)
	Smartphone	69 (46.0)
	Tablet	4 (2.7)

^a^LGBTI: lesbian, gay, bisexual, trans, intersex.

^b^ATSI: Aboriginal and/or Torres Strait Islander.

**Table 3:** Social support levels within the sample.

Support source and nature of support		n	Mean (SD)	Range
**Peer**				
	Positive	147	3.22 (0.63)	1.5-4
	Negative	145	2.42 (0.65)	1-4
**Family**				
	Positive	149	3.31 (0.74)	1-4
	Negative	148	2.96 (0.70)	1-4
**Partner**				
	Positive	44	3.34 (0.77)	1.4-4
	Negative	44	2.03(0.72)	1-3.4

### Likelihood of App Usage

A total of 60.7% (91/150) of participants reported that they were likely to seek help from a mobile app for relationship issues, 26.7% (40/150) had a neutral response, and 12.7% (19/150) were unlikely. Likelihood of app use was not significantly associated with any participant characteristics or social support (all *P* values >.06). Thematic analysis (Table 4) found that 3 key themes influenced participants’ likelihood of use: (1) perceived need, (2) beliefs, and (3) engagement and accessibility.

### Acceptability of App Content

Overall, only 10.6% (16/150) of participants reported that they had not experienced any of the issues presented; that is, most participants (134/150, 89.4%) had experienced 1 or more of the issues presented. All participants reported that their peers would relate to at least one of the characters, with 94.0% (141/150) reporting that their peers would relate moderately or a lot to 1 or more of the characters, and 56.0% (84/150) reporting that their peers would relate a lot to 1 or more of the characters. Table 5 outlines participant responses to the vignettes.

Participants with higher negative family support were more likely to experience Abigail’s issues (*r*_s_=−.25, n=69, *P*=.03), whereas females were more likely than males to report experience of Emily’s issues (*χ*^2^_1_=6.1; OR 5.8, 95% CI 1.50-22.09; *P=*.01). Participants with lower positive family support (*r*_s_=.25, n=69, *P*=.04) and higher negative family support (*r*_s_=−.28, n=69, *P*= *.* 02) were more likely to report experiencing Angus’ issues. No other significant associations were found (all *P* values >.07). On average, 99.3% (149/150) of the sample reported that the proposed suggestions were helpful to some extent. Participants were more likely to rate the advice given for Abigail as helpful if they had some experience of her issues (*r*_s_=.239, n=70, *P*=.05). For Emily, participants were more likely to rate suggestions for her as helpful if they reported higher peer relatedness (*r*_s_=.255, n=70, *P*=.03). Younger participants were more likely to rate suggestions for Jasper as helpful (*r*_s_=−.412, n=70, *P*<.001) as well as Emily’s suggestions (*r*_s_=−.265, n=70, *P*=.03). No other significant associations were found (all *P* values >.43).

**Table 4:** Themes influencing the likelihood of app usage (N=150). R: respondent.

Theme	Definition	n (%)	Example
Perceived need	The degree to which the young person has identified a need for relationship help and support	74 (49.3)	“...I would be able to have a better relationship with my potential girlfriend.” [R132] “I don’t feel I need it at this current stage.” [R2]
Beliefs	The degree to which the young person believed in the effectiveness of mobile apps for providing genuine relationship support	74 (49.3)	“…if it does no harm then it is worth a shot.” [R26] “I’m open to the advice and possibly using such an app, but it also seems a bit silly to use an app for relationship advice.” [R102]
Engagement and accessibility	The degree to which the young person valued the user experience aspects of the app, such as being easy to use, as well as engagement aspects such as being interesting and different	50 (33.3)	“If had useful things and was easily accessible, I would use it. If it was outdated, not useful, hard to interact with etc, I wouldn’t.” [R90] “...if it contains constructive advice and is designed in a way that targets my age group in a positive and welcoming way.” [R64]

**Table 5:** Participant responses to the vignettes (N=150).

Responses		Abigail, n (%)	Jasper, n (%)	Emily, n (%)	Angus, n (%)
**Experience**					
	Yes	79 (52.7)	49 (32.7)	53 (35.3)	27 (18.0)
	No	51 (34.0)	68 (45.3)	65 (43.3)	96 (64.0)
	Unsure	20 (13.3)	33 (22.0)	32 (21.3)	27 (18.0)
**Peer relatedness**					
	A lot	51 (34.0)	23 (15.3)	32 (21.3)	15 (10.0)
	Moderately	68 (45.3)	58 (38.7)	70 (46.7)	53 (35.3)
	A little	27 (18.0)	63 (42.0)	42 (28.0)	76 (50.7)
	Not at all	4 (2.7)	6 (4.0)	6 (4.0)	6 (4.0)
Helpfulness, mean (SD)		4.03 (0.81)	4.12 (0.88)	3.84 (0.89)	3.95 (0.87)

**Table 6:** Themes influencing the acceptability of the proposed relationship-coping strategies (N=150). R: respondent.

Theme	Definition	n (%)	Example
Nature	The degree to which a young person viewed the advice as appropriate, effective, feasible, or credible	127 (84.7)	“It’s very helpful useful information.” [R11] “The advice is theoretically perfect but in reality is very difficult to implement for someone in Emily’s shoes.” [R12]
Scope	The degree to which a young person felt that the advice adequately addressed the full range of issues being faced	38 (25.3)	“There were many elements that I did not imagine, and the points were very comprehensive.” [R68] “You did not address the issue of Emily pressuring her to try marijuana.” [R116]
Approach	The degree to which a young person felt that the advice was nonjudgmental, collaborative, empowering, or condescending	11 (7.3)	“I like that this suggestion understands his reluctance to talk to his parents, or anybody in general, but tries to find ways around that.” [R69] “I think it’s pretty good advice because it doesn’t place any blame on the person receiving it.” [R99]
Personal experience	The degree to which a young person identified personal experience using the advice in the past	10 (6.7)	“I have been in a similar situation and those were pretty close to the steps I took.” [R98] “When I stopped going to school due to my anxiety I did try seeing the school counsellor and they did nothing.” [R10]

Outlined in Table 6, thematic analysis identified 4 key themes that influenced participants’ acceptability of proposed relationship-coping strategies: (1) nature, (2) scope, (3) approach, and (4) personal experience.

When asked what participants would do themselves and recommend to a friend, 8 themes were identified. Outlined in Table 7, 3 themes (seek help, active coping, and perceived coping efficacy) captured participants’ recommendations for what they would do themselves, and 5 themes (general emotional support, informational support, encourage help-seeking, shared activities, and practical support) captured recommendations to a friend facing a relationship issue.

**Table 7:** Participants’ recommendations for what they would do themselves and recommend to a friend when faced with a relationship issue (N=150). R: respondent.

Recommendation type and theme		Definition	n (%)	Example
**What young people would do themselves**				
	Seek help	The degree to which a young person expressed that they would ask for help if faced with a similar issue	64 (42.7)	“Talk to my support teacher to help advise me in the situation.” [R43] “Gain help and advice from trusted friends.” [R66]
	Active coping	The degree to which a young person reported an action-orientated attempt to solve or cope with the problem if faced with a similar issue	50 (33.3)	“Ditch the smoking friend and find better friends.” [R37] “Just try to relax and make myself feel better by doing things I loved.” [R81]
	Perceived coping efficacy	The degree to which a young person felt that they would have the ability to cope if faced with a similar problem	50 (33.3)	“I would tell myself that things get better and try and focus on the positives in life.” [R141] “Not sure, probably withdraw.” [R120]
**What to do to help a friend**				
	General emotional support	This involved acting in a supportive, reassuring, comforting, empathetic, caring, nonjudgmental, and encouraging manner	90 (60.0)	“I would comfort them and make sure they feel loved.” [R117] “I would be there for them in the difficult time that they are going through.” [R110]
	Informational support	This involved providing advice, suggestions, or useful information	61 (40.7)	“Warn her about the effects of marijuana.” [R129] “Convince her to move on and that there are other guys better than Brendan.” [R150]
	Encourage help-seeking	This involved encouraging help-seeking from both formal and informal sources	41 (27.3)	“Encourage them to talk to as professional.” [R1] “I would urge them to see a therapist.” [R80]
	Shared activities	This involved spending time together and engaging in shared activities	22 (14.7)	“Get out and do some sport or hang out.” [R34] “Organize to do things with them to distract them.” [R145]
	Practical support	This involved providing doing something helpful for the friend	19 (12.7)	“I would offer to help her with homework.” [R13]

## Discussion

### Principal Findings

This study aimed to examine young people’s attitudes toward using a mobile phone app for relationship problems and to determine the acceptability of the proposed content. In the current sample, technology ownership was high, with only 1 participant not owning a smartphone or tablet. Almost half of the sample accessed the Internet from their mobile phone. Importantly, two-thirds of the sample indicated that they would be open to using a mobile help-seeking intervention for relationships, irrespective of background factors or levels of social support. These findings suggest that delivering relationship support via a mobile phone app is likely to be accessible to a general youth population and confirms a degree of acceptability for a mobile help-seeking intervention. In terms of future development, several key factors were found to influence the likely use of a mobile app for relationships including perceived need, personal beliefs, engagement, and accessibility. The acceptability of the help-seeking information was influenced by the nature, scope, and approach of the content as well as users’ personal experience of the suggestions. These aspects are likely to be relevant to a range of other youth help-seeking interventions and must be systematically addressed if a mobile intervention is to have broad uptake and appeal.

Notably, likelihood of seeking help from an app was influenced by whether a young person identified a need for relationship support and whether they believed an app would be beneficial. In this study, when asked what they would do when faced with relationship issues, only one-third of the participants reported that they would seek help. Fewer suggested seeking help from a friend. The low level of help-seeking reported by participants aligns with past research on mental health issues, in which youths prefer self-management [19]. These findings are also consistent with depression and suicide, in which individuals reported perceived need as a key driver of help-seeking, alongside personal help-seeking thresholds, beliefs about the usefulness of help-seeking, and trouble identifying symptoms [19,38]. This finding may pose a potential challenge to app developers. For an app to have wide uptake, developers of mobile help-seeking interventions must carefully consider how to address issues of need and usefulness. In the context of the proposed app, a strategy to help users identify areas of need could be the inclusion of screening, in which a user conducts a self-assessment of their relationships to help establish need. However, screening may not always be effective for changing help-seeking behavior [39]. Therefore, it is important that future evaluations of help-seeking apps assess the effectiveness of any functionality aimed at increasing need. In terms of usefulness, positioning an app as a resource that could be used to also help a friend may broaden its appeal because of the importance young people place on relationships. Integration with other youth activities, such as school curriculum or sport, may significantly enhance young people’s knowledge and awareness of mobile help-seeking interventions [40].

Unsurprisingly, young people identified that accessibility and engagement issues, including user experience, influenced their likelihood of using a mobile help-seeking intervention. This is consistent with mobile health app ratings in which users report to value apps that are easy to use, deliver a clear outline of the steps involved to reach a desired goal, and provide personalized information and education tailored to a user’s needs [41]. This can be difficult to achieve in universal programs, and it highlights the importance of considering users’ preferences and contexts early in the design process. Developers must consider content and interface to avoid users’ feeling as though their needs have been disregarded [42,43]. In relation to the proposed app content, all participants reported peer relatedness to at least one of the characters, even though many were uncertain of their own experience of the issues presented. This suggests that the character-driven content has appeal, and as social learning still occurs regardless of similarities [44], the use of character vignettes is likely to be an effective model for changing help-seeking behavior. Furthermore, almost all participants rated the proposed strategies as helpful. Helpfulness was found to be influenced by the general nature of the strategies and whether they adequately addressed the breadth of issues that a young person was experiencing. Interestingly, scheduling shared activities was only suggested by 14% of the sample as a useful strategy, and this may explain why previous apps that focus on behavioral activation may not be effective in this population [30]. A strength of this study is that it enabled end-user assessment of content developed by clinicians and researchers, which Grist and Porter [45] suggest enhances the quality and efficacy of mobile health apps. In addition to an evaluation of effectiveness, next steps will include assessing young people’s views of the proposed app design, including the user interface and app structure, to ensure that the functionality and user experience within the app is positive and engaging.

### Limitations

The current findings must be considered within the study’s limitations. First, this study examined young people’s help-seeking intentions, rather than actual behavior, and may therefore not be a true indication of how young people respond when faced with a relationship problem. Although this is appropriate for acceptability research, future trials would benefit from assessing other objective measures of acceptability, including app usage. Second, the use of an online survey restricted participation only to those who had access to the Internet, and the sample may not have been representative across a range of characteristics. In addition, the sample was recruited primarily from Facebook and Black Dog Institute social media sites. Future studies would benefit from targeting a more diverse sample as this study may have reached a more mental health literate subgroup of youths and those with an interest in mobile apps. Third, it is possible that the suggestions provided in the survey influenced participants’ free responses, thereby creating a learning effect. However, visual inspection of the qualitative data did not indicate participants’ reported help-seeking, coping strategies, or suggestions differed across characters. Finally, a strength of this study is the representativeness of youths who identified as LGBTI, being twice as many than the general population, which is approximately 10% to 11% [46]. It is unclear why this survey achieved such a high level of participation from the LGBTI youth.

### Conclusions

Existing evidence has outlined that young people frequently experience relationship problems and associated distress. The findings of this study substantiate the need for additional relationship support and echo previous research that highlighted young people’s reluctance to seek help from formal services [20,22,23]. This study confirms that youths are likely to use a mobile app that attempts to address these issues. Adolescents in this study were likely to use a mobile phone app for relationship support, and this was not influenced by gender, age, or other background factors. The development of such an app may provide a valuable help-seeking resource for young people. However, acceptability of an app may be increased by addressing the factors of need, personal beliefs, usability, and appropriateness of content. These findings will help to ensure that evidence-based apps have a broad appeal and uptake.

## Appendix

Multimedia Appendix 1 Online survey including the questionnaire, character vignettes, and the app suggestions.

## Abbreviations

ATSI: Aboriginal and/or Torres Strait Islander
CBT: cognitive behavioral therapy
IPT: Interpersonal therapy
LGBTI: lesbian, gay, bisexual, trans, intersex
