# Supplementary material for: Adolescents’ Perspectives on a Mobile App for Relationships: Cross-Sectional Survey
Source: JMIR Mhealth Uhealth. 2018 Mar 8;6(3):e56. doi: 10.2196/mhealth.8831 (PMC5865001; doi:10.2196/mhealth.8831)
Supplement: Multimedia Appendix 1 [file mhealth_v6i3e56_app1.pdf]

Young People and Relationships Study

Welcome to our study! What is this study about?The Black Dog Institute is interested in getting your views about relationships in adolescence and how best to deal with problems. We want to know if and how a mobile phone app can help. This study is being conducted by the Black Dog Institute, at the University of New South Wales. The chief investigator is Dr Bridianne O'Dea.   Who is this study for? This study is only for young people aged 15 - 18 years living in Australia. What does this study involve? This study involves filling out an online survey. This will take up to 30 minutes to finish. All answers are anonymous. You will be asked questions about yourself (like age and gender) and your relationships with friends and family. You will also be asked to read some stories about other young people's relationships and give your advice on what would be helpful for them. At the end, you have the option of receiving a gift voucher for your time ($20AUD). To receive the gift voucher, you will be asked to provide your email and full name in a separate URL. This voucher is only available after full completion of the survey. What happens to the information given in the survey? After you have filled in the survey, your answers will be sent to a secure folder at the University of New South Wales. Only approved researchers will be able to see this data. The researchers will analyse the answers and use this information to design our mobile phone app. All of the survey data will be kept for 7 years. After this time, it will be destroyed.   Do I have to do it? No. Completion is entirely voluntary. If you do not wish to take the survey, you can close this window at any time. Do I have to have my parent's permission? While you do not need to have your parent's permission to take part in the survey, we strongly encourage you to discuss the study with your parents and let them ask any questions they may have.   Are there any risks? We do not think there are any risks to you. If you do become upset, sad, or worried by anything in the survey, we encourage you to talk to a trusted adult (like your parents) or contact Kids Helpline by calling 1800 55 1800. We also encourage you to let the research team know by sending an email to the Chief Investigator b.odea@blackdog.org.au.   What if I want to withdraw? You can stop the survey at any time. However, as the survey is anonymous, once you submit a completed survey we will not be able to withdraw your answers.   How will I find out the results of the study? When the study has finished, we will put a two page summary of the study results on the Black Dog Institute website. We encourage you to visit the website for this update. What if I have questions or complaints? You can contact the chief investigator Dr Bridianne O'Dea by email b.odea@blackdog.org.au. If you want to make a complaint, you can email the our Ethics Office humanethics@unsw.edu.au with the "HC15583" in the subject line.

To take part in this study, please agree with the following:

- ☐ I have read the information above
- ☐ I understand what this study involves
- ☐ I understand that my responses will be analysed and stored for 7 years
- ☐ I don't feel pressured into taking part
- ☐ I am aged 15 - 18 years living in Australia

How old are you?

- ○ 15
- ○ 16
- ○ 17
- ○ 18

Please select your gender

- ○ Male
- ○ Female
- ○ Other

Do you identify as lesbian, gay, bisexual, trans, and/or intersex (LGBTI)?

- ○ Yes
- ○ No
- ○ I'd rather not say

In which country were you born?

- ○ Australia

Other...

....

Do you identify as Aboriginal or Torres Strait Islander?

- ○ Yes
- ○ No
- ○ I'd rather not say

What is the language that you mostly speak at home?

- O English

Other...

....

Are you currently...?

- O In high school
- O Working full time
- O Studying at university
- O Completing an apprenticeship
- O None of the above

Who do you mostly live with?

- O Mum and Dad (Together)
- O Mum and Dad (Separately)
- O Mum only
- O Dad only
- O Other family members
- O Foster parents
- O Other Guardians

Other

....

Do you own a smartphone?

- O Yes
- O No

Do you own a mobile tablet device?

- O Yes
- O No

When acessing the internet, which device do you mainly use?

- ○ My own personal computer (laptop or desktop)
- ○ My own smartphone
- ○ My own mobile tablet
- ○ The family computer (laptop or desktop)
- ○ The family mobile tablet
- ○ A public computer e.g. at school or library

Other...

....

If there was a mobile app to help you have better relationships with your friends, family, boyfriend/girlfriends, how likely are you to use it?

- ○ Extremely unlikely
- ○ Unlikely
- ○ Neutral
- ○ Likely
- ○ Extremely likely

Can you tell us why you would or would not use such an app?

....

....

....

....

How often do your friends...

	Never	Rarely	Sometimes	Often
...make you feel cared for?	○	○	○	○
...express interest in how you are doing?	○	○	○	○
...make too many demands on you?	○	○	○	○
...criticise you?	○	○	○	○
...create tensions or arguments with you?	○	○	○	○

How often do your family...	Never	Rarely	Sometimes	Often
...make you feel cared for?				
...express interest in how you are doing?				
...make too many demands on you?				
...criticise you?				
...create tensions or arguments with you?				

Do you currently have a boyfriend/girlfriend/partner?

- Yes
- No

How much...	Not at all	A little	Some	A lot
...does your partner understand the way you feel about things?				
...can you depend on your partner to be there when you really need them?				
...does your partner show concern for your feelings and problems?				
...can you trust your partner to keep promises to you?				
...can you open up to your partner about things that are really important to you?				
...tension is there between you and your partner?				

	Never	Rarely	Sometimes	Often
How often do you have an unpleasant disagreement with your partner?	○	○	○	○
How often do you things become tense when the two of you disagree?	○	○	○	○
How often does your partner say cruel or angry things during disagreement?	○	○	○	○
How often do the two of you both refuse to compromise during disagreements?	○	○	○	○

We are now going to introduce you to some young people who are going through some relationship problems.   We would like you to read these stories and tell us your thoughts about what would help them right now.

Introducing Abigail   Abigail is 17 and nearly finished school. She studies hard and gets very disappointed when she doesn't get top marks. Abigail likes sport - it relieves her stress. She has had to cut down so she can focus on her school work. Abigail has lots of good friends but usually likes to keep things to herself.   Two months ago, Abigail noticed that she was really starting to like Brendan from her geography class. Normally, Abigail gets very nervous speaking talking to boys on her own. But Brendan was different. She really looked forward to seeing him and felt much happier around him. Abigail didn't tell anyone how she felt. The more time she spent with Brendan, the more she thought he liked her back. But, this all changed when Brendan asked Abigail's best friend, Olivia, out to a movie.   Abigail was very upset by this. She was overtaken by negative thoughts - she blamed her looks and found herself making negative comparisons to Olivia all the time. Abigail felt like she was "fat" and "ugly". Abigail stopped eating and starting losing weight. She knew it wasn't healthy, but she just couldn't stomach food anymore. Sometimes Abigail felt guilty and angry with herself, but at other times she felt relieved – she didn't have control over much in her life except this.

Has this sort of thing happened to you or your friends?

- O Yes
- O No
- O I'm not sure

How much do you think other people your age would relate to Abigail's story?

- O Not at all
- O A little
- O Moderately
- O A lot

If you knew your friend was feeling this way, what would you do to help?

....

....

....

....

What would you do if this happened to YOU?

....

....

....

....

We think the following things might help Abigail get through this tough time: We think that it would be helpful for Abigail to get back into sport, even though she is trying to focus on her school work. It's important to have other things going on in life other than school. We think it would be beneficial for Abigail to learn some practical skills to help her manage negative feelings that do not involve dieting or restricting her food intake. For example, if Abigail could develop some relaxation skills or meditation skills that she could use when she felt 'out-of-control' or very distressed, this will likely calm her down and help her feel more in control. She could try this on her own first (e.g. on her phone using an app), or she could get some professional help (probably by talking to her parents or a trusted adult first). We'd encourage Abigail to speak to a close friend about how she's feeling. She may not feel like talking to Olivia, but since she is well liked and has lots of good friends, she might find it really helpful to open up to someone. Emotions can be a bit like a pressure cooker – if they are kept inside and build and build, they often come out in ways that aren't that helpful (like stopping eating). Often, talking about what is going on can provide a release and stop things building up. If things don't get better for Abigail over the next two weeks, or she loses enough weight that other people start noticing, we strongly encourage Abigail to get some professional help. This might be her school counsellor, or she could speak to a trusted teacher or adult to get some advice on who would be best to help her. If she felt comfortable to talk to her parents about it she could (they might take her to the GP or give her her Medicare card so she could go on her own). If she didn't want to tell her parents, she could go into her local Headspace centre and speak to someone straight away.

On a scale of 1-5, how helpful do you think this advice would be for Abigail?

- ○ 1 = Not at all helpful
- ○ 2 = Slightly helpful
- ○ 3 = Somewhat helpful
- ○ 4 = Very helpful
- ○ 5 = Extremely helpful

Can you tell us why you feel this way?

....

....

....

....

Introducing Jasper Jasper turned 17 last month. The day after his birthday, his first girlfriend Jen broke up with him. Now he feels embarrassed to show up to class, thinking that people are judging him. He had a panic attack when his English teacher asked him to give a report to the class, ending up in the sick bay after feeling faint and dizzy. He has been staying up until 3am playing online games, and has missed school for several days by telling his parents he had stomach problems. He only really feels safe at home, in his bedroom. Jasper is worried about what might happen in Year 12, but doesn't want to talk to anyone about his feelings, least of all his parents. He feels like his parents don't understand what he's going through anyway, and if they tried to help it would just make him feel embarrassed and make his problems worse. After being bullied when he was younger, Jasper finds it hard to trust other people, and thinks he should just take care of his own problems. Things started to change when he met Jen, but now he feels like the future is a bit hopeless. Last weekend, Jasper was playing an online game and formed an alliance with an elf to destroy an evil ogre. He chatted with the elf and found out that she is lives in the same city – her name is Sarah, she is 16 and she goes to the Catholic high school across town. Jasper and Sarah have chatted every night the past week and have arranged to meet up. Jasper is a little anxious about meeting her in person, afraid that she will think he's too nerdy or ugly, even though they have exchanged photos. He doesn't want to be rejected again.

Has this sort of thing happened to you or your friends?

- O Yes
- O No
- O I'm not sure

How much do you think other people your age would relate to Jasper's story?

- O Not at all
- O A little
- O Moderately
- O A lot

If you knew your friend was feeling this way, what would you do to help?

....

....

....

....

What would you do if this happened to YOU?

....

....

....

....

We think the following ideas might help Jasper: We think Jasper should meet up with Sarah in a public place, just to be safe. We would remind Jasper that it's normal to feel nervous when meeting new people, but it's important to remember that he and Sarah have already chatted and exchanged photos, and the likelihood of them not liking each other is low. We think that Jasper can do a few things to make a future panic attack less likely. First of all, sleep is very important for mental health. We would advise Jasper to stop playing computer games late at night. Instead, we would suggest he try going to bed earlier, even if it is just 30 minutes to start. We would encourage Jasper to try practicing the things that make him anxious, so for example, with class presentations, he could practice in front of one friend first or his parents at home. We think that for Jasper to overcome his anxiety and stay well, we need to get the help of trusted adults. We understand that Jasper doesn’t want to speak to his parents, but he should give it a try at least once. If this doesn't work, Jasper might find it useful to talk to one of his teachers at school that he gets on with, or his school nurse or counsellor that he came into contact with in the sick bay. If he really didn’t want to speak to someone in person, he could always have a look online for a program from a reputable and trustworthy organisation like the Black Dog Institute to help reduce anxiety. Once Jasper had some skills to manage his anxiety so that he isn't having panic attacks at school anymore, we think it would be helpful for him to spend time with people he trusts (like Sarah, if it works out). Even if it does work out with Sarah, Jasper should work towards learning to trust other people. It isn’t something that happens automatically, but if he could connect with others (like his online gaming friends), it would mean that he wouldn’t feel so alone.

On a scale of 1-5, how helpful do you think this advice would be for Jasper?

- 1 = Not at all helpful
- 2 = Slightly helpful
- 3 = Somewhat helpful
- 4 = Very helpful
- 5 = Extremely helpful

Can you tell us why you feel this way?

....

....

....

....

Introducing Emily. Emily is about to finish Year 10 at an all-girls high school. Normally she gets good marks, but last month she failed her first exam ever. She was too scared to tell her parents because she knew they would freak out. Her parents had already gotten a phone call from the school about her marks getting worse, and it has made things bad at home – her parents yelled a lot and fought with her. This wasn't a surprise for Emily because home is a pretty stressful place. Her parents often fight. Emily works a part-time job which is a distraction from her home life. Recently, Emily had a fight with one of her friends Joey. Joey is now being very weird about it and Emily's whole friend group has been taunting her in the hallways and sending her text messages about how she is no longer part of the group. Emily is upset and confused. Lately, it has all become too much for Emily and she has been skipping school with a new girl, Alex. Alex seems to get her. This is partly because Alex's parents are getting divorced. Emily finds Alex easy to talk to and felt she could be herself around Alex. The one problem with their relationship is that Alex smokes marijuana pretty regularly and has been encouraging Emily to try it. Emily has an uncle with psychosis, so knows she shouldn't do it but likes the idea of escaping her problems. She also wants to do it to feel closer to Alex. Emily feels like she has a deeper and deeper connection with her and is starting to wonder if she may like her as more than just a friend.

Has this sort of thing happened to you or your friends?

- O Yes
- O No
- O I'm not sure

How much do you think other people your age would relate to Emily's story?

- O Not at all
- O A little
- O Moderately
- O A lot

If you knew your friend was feeling this way, what would you do to help?

....

....

....

....

What would you do is this happened to YOU?

....

....

....

....

Here are our ideas for what might help Emily: We think Emily should tell her parents about her failed exam. Even though it might be tough, we think it’s better to come from her. We think it might help to have another adult there, such as a trusted teacher or a school counsellor. Together, they can help Emily to come up with a plan to get her grades back on track. We think it’s also time to take action on her parent's fighting. We would suggest that Emily could try talking to one or both of her parents (when they are calm) about how much the fighting upsets her, and that it makes her afraid that they’re going to get a divorce. If that seems too difficult, we think Emily needs to speak to another family member, a counsellor, or a doctor to get their support. It’s important Emily knows that her parents’ fights are not her fault. We also think would suggest that Emily find a quiet place to go to when her parents are fighting – like her room with headphones on, or somewhere out in the back yard where she can get away from the fighting. We think that Emily would benefit from learning the skills to challenge her negative thoughts, and to think more helpfully about what is going on in her life right now. This usually requires some help, so we would suggest she see her school counsellor, or doctor, or psychologist who can help her. We also think that Emily’s feelings for Alex might be genuine, or they might be more about valuing Alex’s friendship at a time when she is feeling lonely and misunderstood. We would suggest to Emily to try to focus on resolving her fights with her friends and if these feelings are still there after that, suggest that Emily seek out reliable information about sexual orientation from trusted sources like Headspace, or her School Counsellor.

On a scale of 1-5, how helpful do you think this advice would be for Emily?

- ○ 1 = Not at all helpful
- ○ 2 = Slightly helpful
- ○ 3 = Somewhat helpful
- ○ 4 = Very helpful
- ○ 5 = Extremely helpful

Can you tell us why you feel this way?

....

....

....

....

Introducing Angus: Angus is 16 years old and feels hard done by. He was made to transfer to boarding school in a new city when his parents separated. He left his old school, his community, his friends, and his life. He arrived in a new school and a new place full of annoying kids with stuck up parents. He feels he is the last virgin left in NSW and he has never really ever kissed a girl. Angus is especially angry with his mum whom he expected to stay in her job in the other city till he finished school. He really resents his little brother John – his parents think that John is the 'easy' one and Angus is the 'difficult' one. John wasn't made to go to boarding school when they moved, and Angus almost hates him for that. On the weekends, Angus just ignores John. One weekend, Angus goes back to his home town by himself and picks some of his own buddies to come over unsupervised and drink beer. He used to love hanging out with his mates – he misses the fun and the clowning around. Angus is normally a bit of a joker and he loves being able to make people laugh. Angus also misses his old football team. He's just started playing at the new school, but even on the field he's having trouble controlling his anger. Angus has always been a bit fiery, but not like this. His coach pulled him aside the other day and asked him how he was settling in with the other boys, and said that he'd noticed Angus was getting a bit 'fired up' during the game. The coach is about the only person who has taken any interest in him but it's hard to talk to anyone about what he's going through. No one else really cares anyway. It's just so unfair – he can't stop asking why did his parents have to split up? And why is he the only one who had to go to boarding school?

Has this sort of thing happened to you or your friends?

- Yes
- No
- I'm not sure

How much do you think other people your age would relate to Angus' story?

- Not at all
- A little
- Moderately
- A lot

If you knew your friend was feeling this way, what would you do to help?

....

....

....

....

What would you do if this happened to YOU?

....

....

....

....

Here are our ideas for what might help Angus: We think it would help Angus to know that moving schools and cities is often hard at first, but does get easier. We would suggest Angus try to make friends through the school football team, even though he doesn't think he is similar, he might find that at least some of them are okay. We think that Angus' football coach could be a good person to turn to. Rather than bottling up all of his thoughts and feelings, Angus could make some time to talk to his coach about what's bothering him – sometimes it's easier to talk to someone who is removed from family problems. We think that Angus should try to find a way to let his parents know how difficult this is for him, but he'll need to do it in an assertive way rather than an aggressive way so that his message gets through. He could try telling his mum that he's found it really hard to leave his old friends and that he wishes they hadn't left. This might lead to a more helpful conversation with mum. He could also ask his mum to help arrange a visit to go back to see his old mates in the school holidays. We think that Angus could also use some help to change how he is thinking about his situation. At the moment, he seems to be seeing things as all bad. A more balanced way to think about it might be to acknowledge that things are tough right now, but also that he has some options to make it better.

On a scale of 1-5, how helpful do you think this advice would be for Angus?

- ○ 1 = Not at all helpful
- ○ 2 = Slightly helpful
- ○ 3 = Somewhat helpful
- ○ 4 = Very helpful
- ○ 5 = Extremely helpful

Can you tell us why you feel this way?

....

....

....

....

You have now completed the survey! To submit your answers, please click the submit button below. You will then be re-directed to a secure website where we will collect your email and name to send you the e-voucher. Thank you for your time!
